# Complete Genome Sequences of Group III *Campylobacter* Bacteriophages PC5 and PC14

**DOI:** 10.1128/genomeA.01030-16

**Published:** 2016-11-10

**Authors:** Nika Janež, Matjaž Peterka, Tomaž Accetto

**Affiliations:** a Centre of Excellence for Biosensors, Instrumentation and Process Control, Centre for Biotechnology, Ajdovščina, Slovenia; b University of Ljubljana, Biotechnical faculty, Animal Science Department, Domžale, Slovenia

## Abstract

Here, we present the whole-genome sequences of bacteriophages PC5 and PC14 specific for *Campylobacter jejuni*, a leading cause of gastroenteritis in developed countries. Their genomes are syntenic to those of group III *Campylobacter* bacteriophages and share more than 90% identity at the nucleotide level with members of this group.

## GENOME ANNOUNCEMENT

An increasing number of gastrointestinal infections caused by *Campylobacter* spp. in the developed world prompted the exploration of bacteriophages as potential tools to decrease levels of contamination with these bacteria in poultry production (1). *Campylobacter*-specific bacteriophages PC5 and PC14 belong to the *Myoviridae*, as determined by electron microscopy (2). They were recovered from chicken ceca in Slovenia in 2011 (2). The bacteriophages were propagated using a plate lysis method, and DNA was extracted as described earlier (2). DNA was sequenced by 454 pyrosequencing (GS FLX; Roche). Sequences were assembled using Newbler software and annotated by the Prokka annotation pipeline (3) using a custom blast database for homology search, which consisted of proteins annotated in the *Campylobacter* bacteriophages CPX, CP30A, and NCTC12673.

The genome sizes were 131,095 bp and 134,927 bp for PC5 and PC14, respectively. The G+C contents of both genomes are 26%. The nature of the genome termini was predicted from comparative analysis of the large terminase subunit translated gene sequence (4). The putative large terminase subunits of PC5 and PC14 clustered together with T4 and related bacteriophages. These bacteriophages are therefore likely to form DNA ends similar to T4 headful packaging bacteriophages.

Ninety-four percent of the genomes are made up of coding sequences. One hundred seventy-two open reading frames were identified in PC5 and 171 open reading frames in PC14, while both genomes also code for 3 tRNAs. A set of genes associated with DNA metabolism (for replication, putative topoisomerase, sliding clamp, and single-stranded DNA [ssDNA] binding protein; for repair and recombination, exonuclease; and for packaging, terminase) and virion proteins (capsid, neck, and tail components) was identified using blastp and HMMER. The level of conservation between PC5 and PC14 is very high, as they share 97% identity over 95% of the sequence at the nucleotide level. The majority of matches from database sequences belong to other *Campylobacter* group III bacteriophages, and they share more than 90% identity at the nucleotide level; homologues were found also among cyanobacteriophages of *Prochlorococcus* and *Synechococcus*. In the PC5 genome, there are 16 unique coding sequences (CDSs), and there are 13 unique CDSs within the PC14 genome. The majority of these do not have significant matches in database sequences.

A mainly chitin-specific carbohydrate binding module 5 (CBM5) protein was found (PC5, positions 704 to 1222; PC14, positions 49240 to 49758); additionally, in PC5 only, a glycoside hydrolase 23 (GH23) domain (positions 8853 to 9419) containing a protein, a putative lysin, was found. An intein-like sequence was found within putative ribonucleotide reductase from PC14 but not in PC5. The three copies of methylase domain proteins identified in PC14 were found also within the PC5 genome and other *Campylobacter* group III bacteriophages. The PC5 genome additionally contains two unique proteins with methylase domains, PC_138 (positions 97251 to 97529) and PC5_139 (positions 97492 to 98062), similar to bacterial methylases.

Putative homing endonucleases and their homologues were found in 11 and 12 copies within PC5 and PC14, respectively. The three tRNA genes in both genomes are tRNA-Met, tRNA-Asn, and tRNA-Tyr.

### Accession number(s).

The complete whole-genome sequences were deposited in GenBank under accession numbers KX229736 (PC5) and KX236333 (PC14).
